# Construction of a novel clinical nomogram to predict cancer-specific survival in patients with primary malignant adrenal tumors: a large population-based retrospective study

**DOI:** 10.3389/fmed.2023.1184607

**Published:** 2023-05-25

**Authors:** Mingzhen Li, Xiaoying Duan, Di You, Linlin Liu

**Affiliations:** ^1^Department of Radiation Oncology, China-Japan Union Hospital of Jilin University, Changchun, China; ^2^Department of Acupuncture and Moxibustion, Second Hospital of Jilin University, Changchun, China; ^3^Department of Anesthesiology, China-Japan Union Hospital of Jilin University, Changchun, China

**Keywords:** adrenal tumor, Cancer-specific survival, nomogram, risk stratification system, SEER

## Abstract

**Background:**

Primary malignant adrenal tumors were rare and had a poor prognosis. This investigation aimed to create a useful clinical prediction nomogram to anticipate cancer-specific survival (CSS) of patients with a primary malignant adrenal tumor.

**Method:**

This study included 1748 patients with malignant adrenal tumor diagnoses subjects from 2000 to 2019. These subjects were allocated randomly into training (70%) and validation (30%) cohorts. Patients with adrenal tumors underwent univariate and multivariate Cox regression analyses to identify the CSS-independent predictive biomarkers. Therefore, a nomogram was created depending on those predictors, and calibration curves, receiver operating characteristic (ROC) curves, and decision curve analysis (DCA) were used to assess the calibration capacity of the nomogram, discriminative power, and clinical efficiency, respectively. Afterward, a risk system for categorizing patients with adrenal tumors was established.

**Result:**

The univariate and multivariate Cox analysis demonstrated the CSS-independent predictive factors, including age, tumor stage, size, histological type, and surgery. As a result, a nomogram was developed using these variables. For the 3-, 5-, and 10-year CSS of this nomogram, the values of the area under the curve (AUC) of the ROC curves were 0.829, 0.827, and 0.822, respectively. Furthermore, the AUC values of the nomogram were higher than those of the individual independent prognostic components of CSS, indicating that the nomogram had stronger prognostic prediction reliability. A novel risk stratification method was created to further improve patient stratification and give clinical professionals a better reference for clinical decision-making.

**Conclusion:**

Through the developed nomogram and risk stratification method, the CSS of patients with malignant adrenal tumors could be predicted more precisely, assisting physicians to differentiate patients better and creating personalized treatment strategies to optimize patient benefits.

## 1. Introduction

The adrenal gland is one of the most important endocrine organs that synthesize and secrete hormones in the body (1). Adrenal incidentalomas (AI) are relatively common. The average prevalence rate of AI in autopsy is about 2%, which increases with age and does not show significant differences between men and women (2). The prevalence of AI in the middle-aged population is approximately 4%. It is lower in children and adolescents, accounting for about 0.3 to 0.4% of all tumors in children, but can reach up to 10% in senior citizens (3, 4). However, only a tiny proportion of AI is malignant (< 2%), with the most common histological type being adrenocortical carcinoma (ACC) and neuroblastoma (NB) (5). The most common malignant tumor of the adrenal gland in adults is ACC (6), with a worldwide incidence of 0.5–2.0 per million people yearly, while the estimated US incidence of ACC is 0.72 (7). The prognosis for patients with ACC is usually poor, with a 5-year survival rate of approximately 16–38% (8). In children, the most common adrenal malignancy is NB, with an incidence of 1/7000 to 1/8000 (9). Each year, 700 cases of NB are newly diagnosed in the United States, and approximately 15% of all pediatric cancer-related deaths are due to NB (10, 11). NB is a heterogeneous disease with different prognoses depending on age at diagnosis, stage, tumor size, and biological features of individual patients (12). For high-risk NB children, the 5-year survival rate remains below 50% (13).

Surgical tumor removal remains the primary treatment for malignant adrenal tumors (14). Adjuvant radiotherapy and chemotherapy may also be administered to patients with advanced or recurrent adrenal tumors (12, 15). Additionally, there have been some studies on immunotherapy and targeted therapy for adrenal tumors, but whether they could improve the prognosis of patients with adrenal tumors remains controversial. Therefore, it is essential to perform distinct research to discover the highest significant prognostic indicators linked to the survival rate of subjects with adrenal tumors and to implement individualized therapy for those individuals.

Nomogram is a multi-metric combined model that can predict disease occurrence or progression in tumor survival prediction. It is employed to assess and anticipate the survival of cancer patients and help make better therapeutic decisions (16). Additionally, it was admitted into the National Comprehensive Cancer Network guideline as a robust tool to facilitate personalized clinical prediction (17). The benefit of a nomogram is that it may simplify a complicated statistical prediction with many factors in one anticipating model that is numerically quick to estimate the chance of an occurrence. Compared to overall survival (OS), cancer-specific survival (CSS) offers more accurate therapy recommendations with a closer correlation with tumor-mediated patient prognosis. Consequently, we aimed to develop a predictive nomogram suitable for primary malignant adrenal tumor patients with different histological types, including ACC, NB, pheochromocytoma (PHEO), and other rare adrenal malignancies. CSS was identified as the endpoint of this study. We then set out to discover independent prognostic markers related to CSS in malignant adrenal tumor patients by evaluating data from the surveillance, epidemiology, and end results (SEER) database, thereby creating a novel nomogram together with a risk categorization system for the 3-, 5-, and 10-year CSS anticipation regarding adrenal tumor subjects.

## 2. Materials and methods

### 2.1. Database

The US population database SEER^1^ contains 17 cancer registries, covering about 30% of the US population, offering proof to support medical practice and study throughout the globe (18). For all participants with adrenal malignancies in the SEER database from 2000 to 2019, SEER Stat 8.4.1 was used to retrieve the pertinent data under reference number 17720-Nov2021 [Incidence-SEER Research Plus Data, 17 Registries, Nov 2021 Sub (2000–2019)]. The informed consent or authorization from the ethics committee of the patient was unnecessary since SEER is open to the public, and no specific personal information was included in the data obtained. This study complies with STROCSS 2021 standard (19).

### 2.2. Patient selection

The criteria for inclusion were: (1) the main patient primary tumor sites were the adrenal cortex (C74.0), the adrenal medulla (C74.1), and the adrenal gland, NOS (C74.9), (2) a clear ICD-O-3 histological type of the patient, (3) primary tumor, (4) complete and detailed follow-up information, and (5) the death identification of patient was “live or dead from cancer.” The exclusion criteria were: (1) not the primary malignancy, (2) data on age, gender, race, marital status, tumor size, stage, surgery, radiotherapy, chemotherapy, along with ICD-O-3 histology/behavior were unknown, and (3) a survival period of lower than 1 month or unknown. Finally, 1748 patients participated in this study and were categorized randomly with a 7:3 ratio into the training (*n* = 1,224, 70.0%) and validation (*n* = 524, 30.0%) sets (20). On the bias of prognostic variables, the training cohort was examined for independent prognostic indicators along with the creation of a nomogram and a new evaluation system for patients’ risk, which were verified regarding their predictive reliability and accuracy via the validation set.

### 2.3. Variable definition

Herein, the following variables were employed: (1) demographics of subjects (age, sex, race, marital status), (2) disease characteristics (tumor ICD-O-3 histology/behavior (histological type), tumor size, and tumor stage), and (3) management information (surgery, radiotherapy, chemotherapy, and surgery/radiation sequence). The X-tile software helped determine the ideal age cut-off values for the adrenal tumor patients, and the analysis indicated that 9 and 61 years old were the best cut-off values for age. Moreover, using the X-tile program, 63 and 111 mm were the best thresholds for tumor size. The race categories were white, black, and others; the sex was male or female. Marital status was categorized as either unmarried or married. Chemotherapy, radiation, and surgery were categorized as yes or no. The cancer stage categorization was localized, regional, and distant. The histological type was divided into NB, ACC, PHEO, and others. Our trial’s primary endpoint, or CSS, was from the diagnosis day to the death day caused by this cancer (alive or dead due to cancer).

### 2.4. Data analysis

R (4.2.1) and SPSS (27.0) software deserve credit for all data analysis that was done, and a *p*-value of <0.05 was regarded as statistically significant. First, specified values were assigned individually to each variable, and a table was created containing crucial epidemiological and clinicopathological traits of patients with malignant adrenal tumors. Second, the statistically relevant factors were then determined as a consequence of each variable’s univariate and multivariate Cox regression analysis. Third, the nomogram was built on the basis of the acquired independent predictors to predict the patient’s 3-, 5-, and 10-year CSS. Additionally, to prove the nomogram’s calibration accuracy and discriminatory ability, the calibration and receiver operating characteristic (ROC) curves were developed. Consequently, the clinical application value was measured by decision curve analysis (DCA). Furthermore, scores for each predictive component were added to calculate the overall risk scores of the patients, and the most appropriate cut-off value of the risk score was determined by the X-tile (4.2.1) software. Finally, using risk scores, patients were divided into high-, middle-, and low-risk subgroups, and variations in CSS between different risk groups of patients were demonstrated by the KM curves.

## 3. Results

### 3.1. Patient characteristics

As the primary data source for our experiment, 1748 patients with adrenal malignancies from the SEER database were enlisted. Using the R software, we randomly assigned the patients to the training (*n* = 1,224, 70.0%) and validation (*n* = 524, 30.0%) groups using a 7:3 ratio. Most patients with malignant adrenal tumors were white (n = 1,399, 80.0%), with diagnosis age < 9 years (*n* = 746, 42.7%), unmarried (*n* = 1,174, 67.2%), and sex did not have significant differences. The most prevalent tumor stage and size were distant metastasis (*n* = 863, 49.4%) and size 63–111 mm (*n* = 649, 37.1%). Regarding the histology type, NB (*n* = 761, 43.5%) was more prevalent than ACC (*n* = 693, 39.7%), PHEO (*n* = 151, 8.6%), and other (*n* = 143, 8.2%). Regarding treatment, 82.6% received surgery, 24.3% underwent radiotherapy, 53.6% received chemotherapy, and only 20.7% received radiation after surgery (Table 1).

**Table 1:** The demographics and clinicopathologic characteristics of patients with adrenal malignancies.

Variables	Training cohort		Validation cohort		Total	
	1,224	70.0%	524	30.0%	1748	100%
Age (years)						
<9	521	42.6%	225	42.9%	746	42.7%
9–61	481	39.3%	203	38.8%	684	39.1%
>61	222	18.1%	96	18.3%	318	18.2%
Race						
Black	157	12.8%	51	9.7%	208	11.9%
White	962	78.6%	437	83.4%	1,399	80.0%
Other	105	8.6%	36	6.9%	141	8.1%
Sex						
Male	583	47.6%	261	49.8%	844	48.3%
Female	641	52.4%	263	50.2%	904	51.7%
Marital status						
Unmarried	810	66.2%	364	69.5%	1,174	67.2%
Married	414	33.8%	160	30.5%	574	32.8%
Tumor stage						
Localized	417	34.1%	150	28.6%	567	32.4%
Regional	212	17.3%	106	20.2%	318	18.2%
Distant	595	48.6%	268	51.2%	863	49.4%
Tumor size (mm)						
<63	408	33.4%	166	31.7%	574	32.9%
63–111	446	36.4%	203	38.7%	649	37.1%
>111	370	30.2%	155	29.6%	525	30.0%
Histological type						
Neuroblastoma	524	42.8%	237	45.2%	761	43.5%
Adrenocortical carcinoma	491	40.1%	202	38.5%	693	39.7%
Pheochromocytoma	104	8.5%	47	9.0%	151	8.6%
Other	105	8.6%	38	7.3%	143	8.2%
Surgery						
No	194	15.8%	111	21.2%	305	17.4%
Yes	1,030	84.2%	413	78.8%	1,443	82.6%
Radiation						
No	924	75.5%	400	76.3%	1,324	75.7%
Yes	300	24.5%	124	23.7%	424	24.3%
Chemotherapy						
No	573	46.8%	238	45.4%	811	46.4%
Yes	651	53.2%	286	54.6%	937	53.6%
Surg/Rad.Seq						
Radiation after Surgery	254	20.8%	108	20.6%	362	20.7%
Other	970	79.2%	416	79.4%	1,386	79.3%

Surg/Rad.Seq, Surgery/Radiation.Sequence.

### 3.2. Independent predictive variables for CSS

The recognition of CSS-independent predictive factors in adrenal tumor patients via the uni-and multivariate Cox regression analyses. The univariate Cox analysis assessed the following 11 factors: age, race, sex, marital status, tumor size, stage, histological type, surgery, radiotherapy, chemotherapy, and surgery/radiation sequence. Age, tumor stage, tumor size, histological type, marital status, chemotherapy, and surgery were considered CSS-related (*p* < 0.05). However, race, sex, radiotherapy, and surgery/radiation sequence did not show statistically significant differences (*p >* 0.05). Finally, the results of the multivariate Cox analysis demonstrated that CSS-independent prognostic risk factors were as follows: diagnosis age > 61 years (*p* < 0.001), distant tumor metastasis (*p* < 0.001), tumor size >111 mm (*p* < 0.001), as well as the histological type of ACC (*p* = 0.015). At the same time, the CSS-independent protective prognostic factor was surgery (*p* < 0.001) (Table 2).

**Table 2:** The univariate and multivariate Cox analyses results.

Variables	Univariate analysis OR (95% CI)	*p*-value	Multivariate analysis OR (95% CI)	*p*-value
Age (years)				
<9	Reference		Reference	
9–61	2.761 (2.25–3.388)	≤0.001	2.925 (1.873–4.568)	≤0.001
>61	4.525 (3.611–5.671)	≤0.001	5.103 (3.175–8.202)	≤0.001
Race				
Black	Reference			
White	1.209 (0.938–1.559)	0.144		
Other	1.125 (0.776–1.631)	0.535		
Sex				
Male	Reference			
Female	0.944 (0.803–1.111)	0.489		
Marital status				
Unmarried	Reference			
Married	2.074 (1.762–2.442)	≤0.001		
Tumor stage				
Localized	Reference		Reference	
Regional	1.696 (1.304–2.206)	≤0.001	1.814 (1.391–2.365)	≤0.001
Distant	2.673 (2.184–3.271)	≤0.001	4.36 (3.465–5.485)	≤0.001
Tumor size (mm)				
<63	Reference		Reference	
63–111	2.385 (1.903–2.988)	≤0.001	1.566 (1.244–1.972)	≤0.001
>111	3.157 (2.518–3.959)	≤0.001	1.651 (1.294–2.107)	≤0.001
Histological type				
Neuroblastoma	Reference		Reference	
Adrenocortical carcinoma	3.457 (2.844–4.204)	≤0.001	1.736 (1.113–2.706)	0.015
Pheochromocytoma	1.11 (0.761–1.62)	0.587	0.667 (0.383–1.161)	0.152
Other	3.914 (2.953–5.186)	≤0.001	1.568 (0.955–2.574)	0.076
Surgery				
No	Reference		Reference	
Yes	0.323 (0.267–0.391)	≤0.001	0.551 (0.444–0.682)	≤0.001
Radiation				
No	Reference			
Yes	1.028 (0.854–1.238)	0.77		
Chemotherapy				
No	Reference			
Yes	1.335 (1.132–1.575)	0.001		
Surg/Rad.Seq				
Radiation after Surgery	Reference			
Other	1.203 (0.981–1.477)	0.076		

OR, odds ratio; CI, confidence interval; Surg/Rad.Seq, Surgery/Radiation.Sequence.

### 3.3. Establishing and verifying prognostic nomogram for CSS

**Figure 1:**
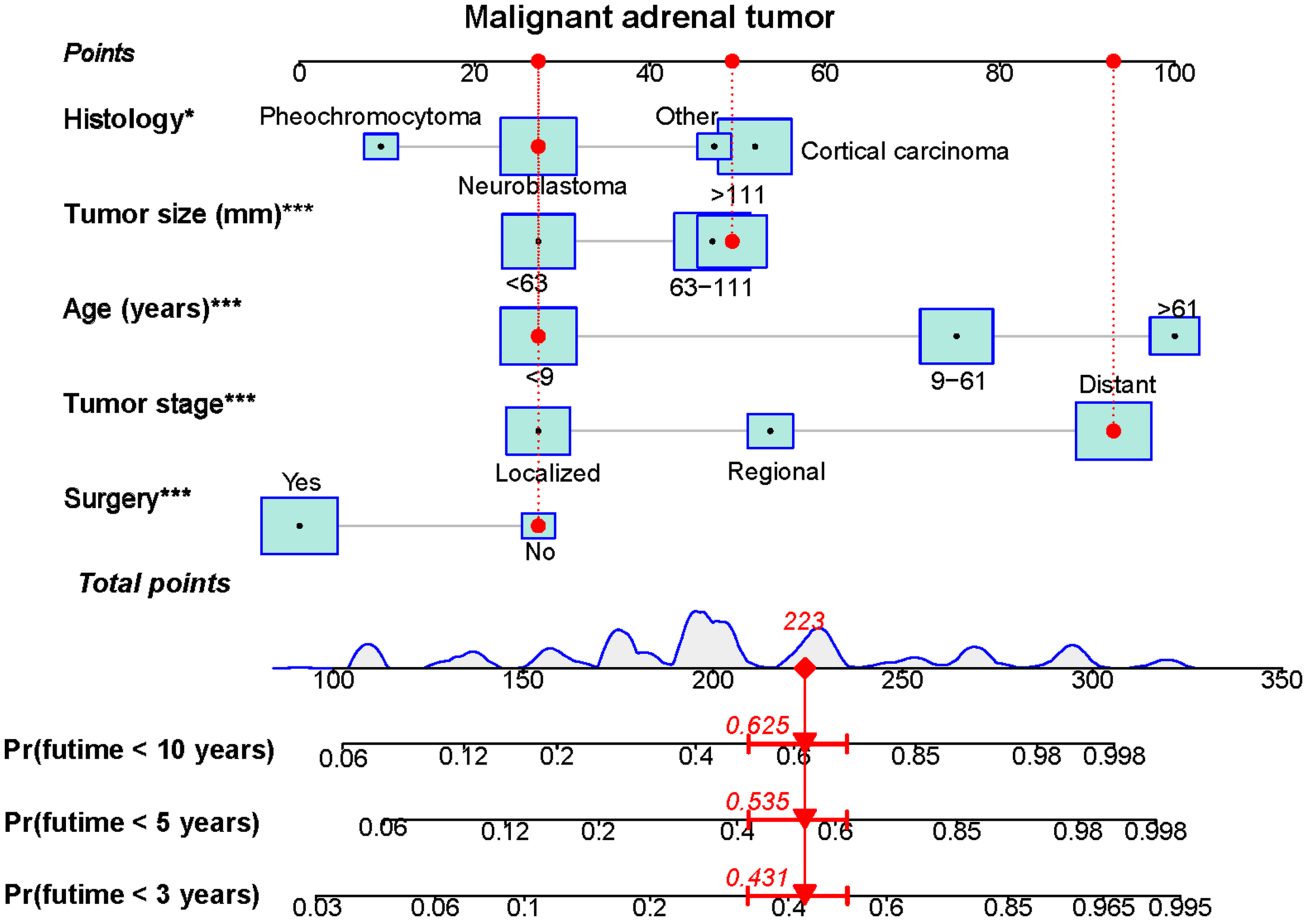
A prognostic nomogram was constructed to predict 3-, 5- and 10-year CSS of malignant adrenal tumor patients (*p<0.05, ***p<0.001). Specifically, when a patient with primary malignant adrenal tumor consults about his or her survival, we can sum the points of independent prognostic factors to obtain a total score and draw a vertical line from the total points to the bottom timeline to obtain his or her 3-, 5-, and 10-year death probability. The probability of survival at the corresponding time can be obtained by subtracting the probability of death from 1. For example, a 7 years old patient diagnosed with a 125 mm distant metastasis primary malignant neuroblastoma tumor received no surgery. The corresponding total points of his or her is 27 (7 years old) + 49 (125 mm size) + 93 (distant metastasis) + 27 (neuroblastic tumor) + 27 (without surgery) = 223, and the corresponding death probability at 3, 5, and 10 years are 0.431, 0.535, and 0.625, respectively, while the patient’s corresponding probability of CSS at 3, 5, and 10 years are 0.569, 0.465, and 0.375, respectively.

**Figure 2:**
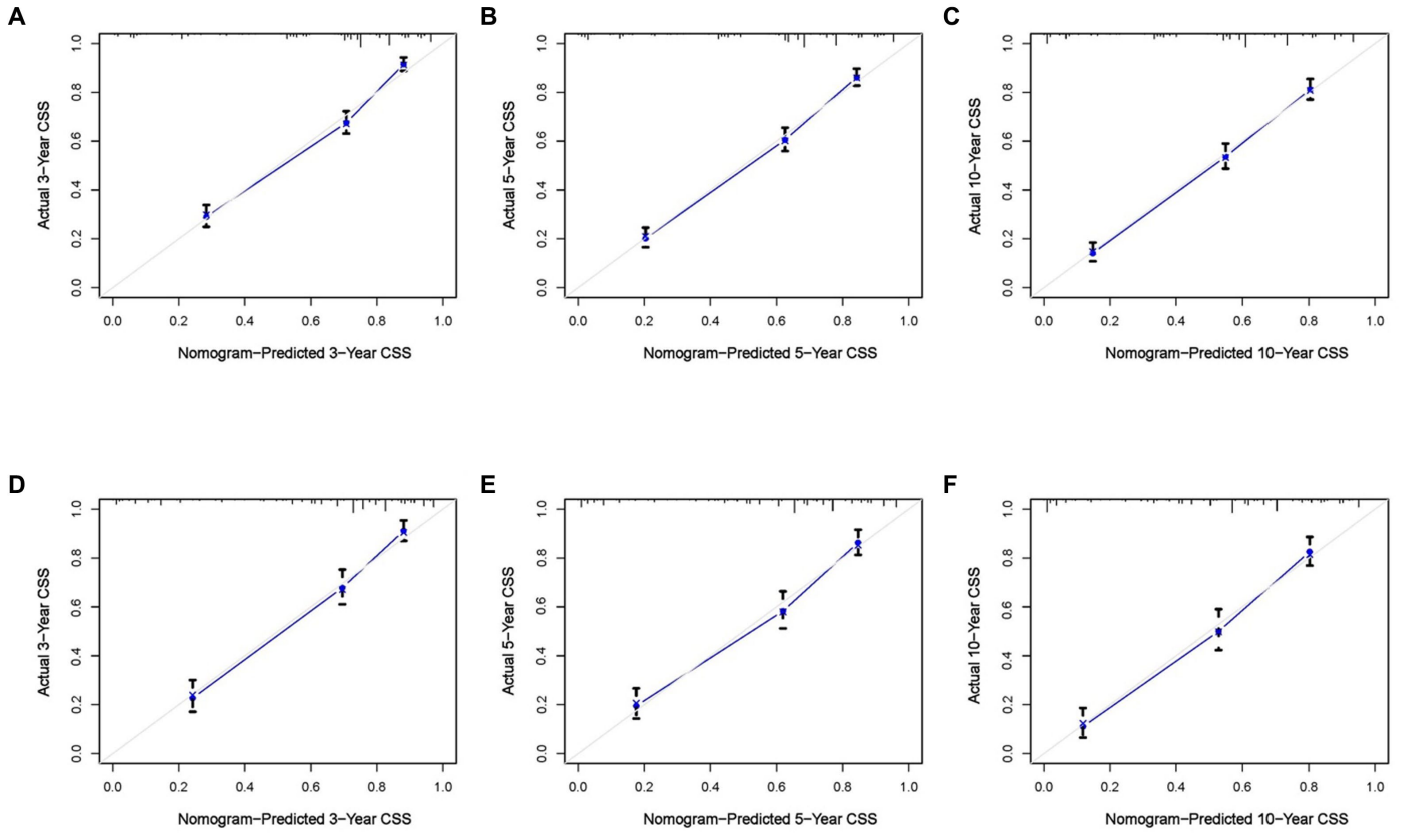
Calibration curves of the constructed nomogram at 3 **(A)**, 5 **(B)**, and 10 **(C)** years CSS of malignant adrenal tumor patients in the training cohort and 3 **(D)**, 5 **(E)**, and 10 **(F)** years in the validation cohort. The X-axis represents the nomogram-predicted CSS rate, whereas the Y-axis represents the actual CSS rates in this study.

**Figure 3:**
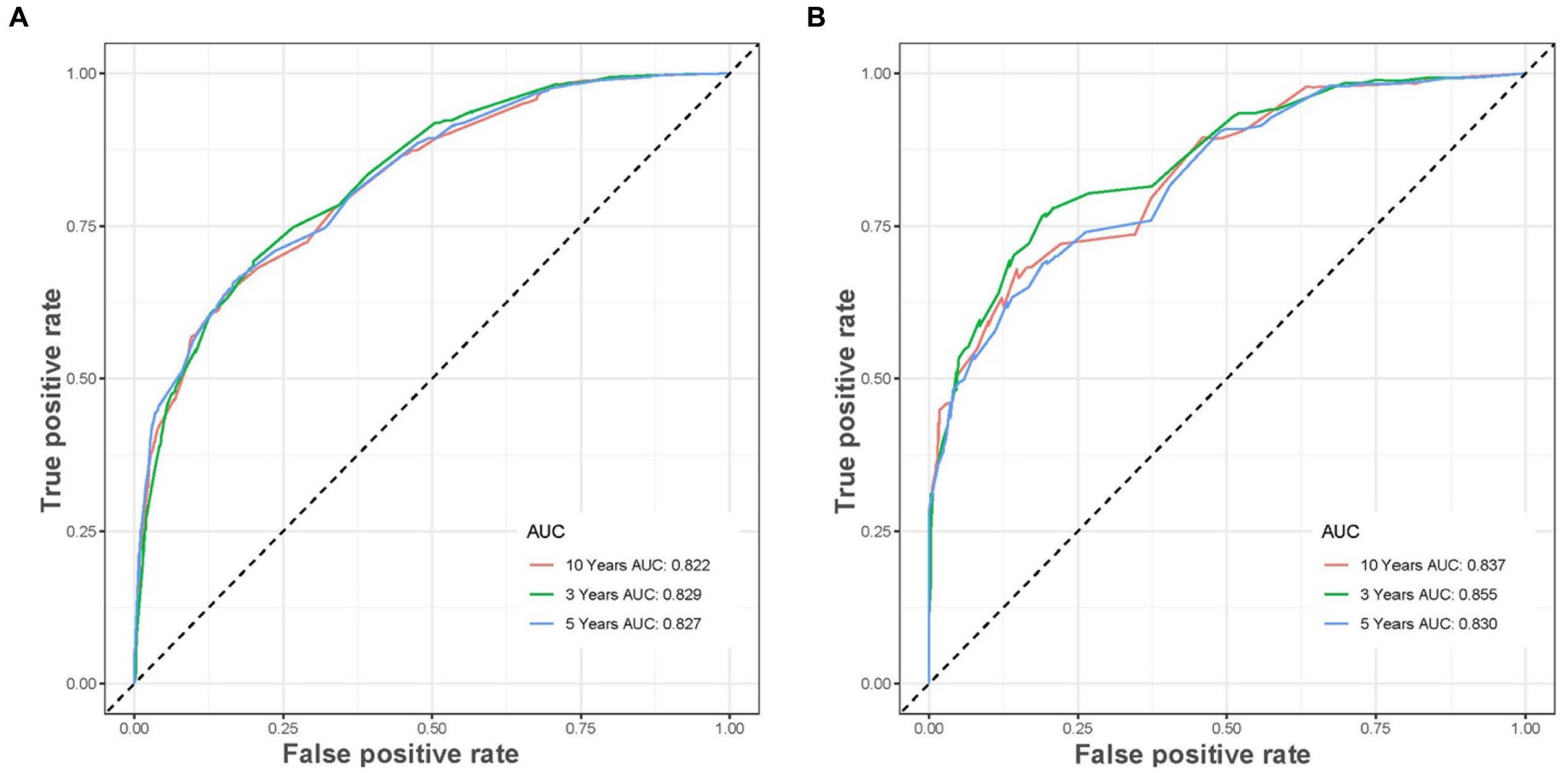
The receiver operating characteristic curves of CSS prediction of malignant adrenal tumor patients at 3, 5, and 10 years in the training **(A)** and validation **(B)** cohorts.

**Figure 4:**
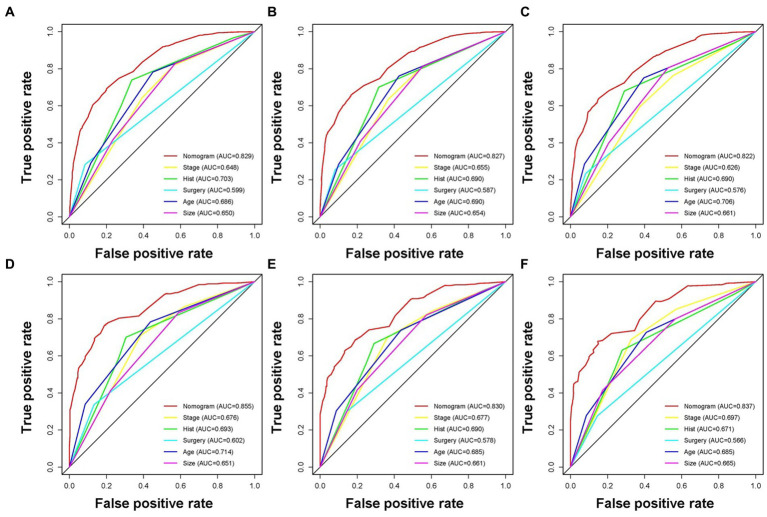
The receiver operating characteristic curves compare the prediction accuracy between the constructed nomogram and each CSS-related independent predictor of malignant adrenal tumor patients at 3 **(A)**, 5 **(B)**, and 10 **(C)** years in the training cohort and 3 **(D)**, 5 **(E)**, and 10 **(F)** years in the validation cohort.

**Figure 5:**
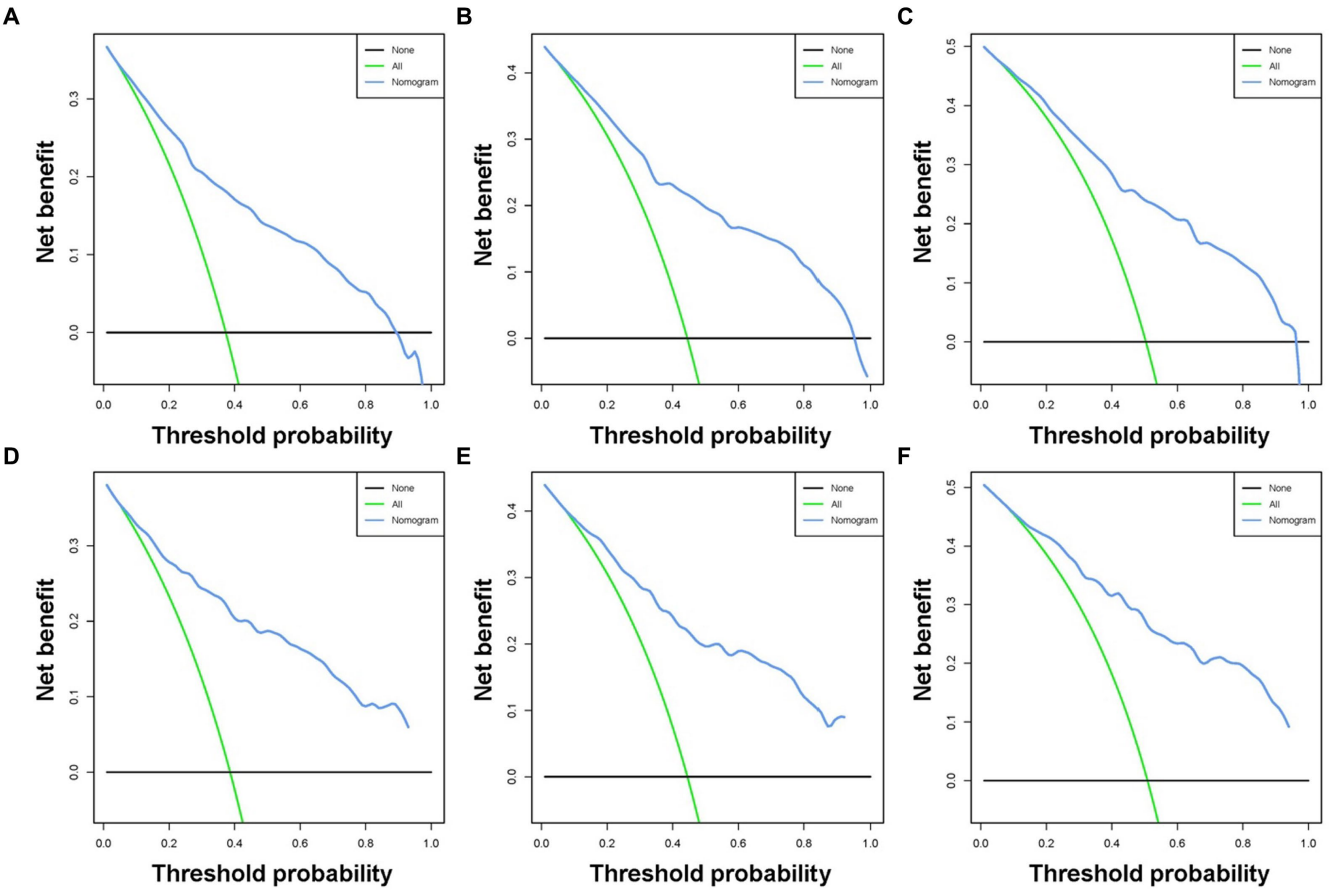
Decision curve analysis was used for predicting the 3-**(A)**, 5-**(B)**, and 10-**(C)** year CSS probability of malignant adrenal tumor patients in the training cohort and the 3-**(D)**, 5-**(E)**, and 10-**(F)** year CSS probability in the validation cohort.

Based on the five independent prognostic variables, a predictive nomogram was developed to anticipate the CSS regarding adrenal tumor patients in a quantitative way (Figure 1). After drawing a vertical line to the nomogram’s first row, we determined the scores that corresponded to each of the independent factors. It indicated that the prognosis was generally poor for patients with age > 61 years, distant metastases, tumor size >111 mm, ACC, and did not receive surgery. According to the calibration curves, there is a satisfactory correlation between the patient’s actual rates of 3-, 5-, and 10-year CSS and the anticipated rates obtained from the generated nomogram (Figure 2). The training set’s AUCs for the 3-, 5-, and 10-year CSS were 0.829, 0.827, and 0.822, respectively, while for the validation set, they were 0.855, 0.830, and 0.837, correspondingly (Figure 3). Moreover, the predictive accuracy of the generated nomogram was compared to that of each independent predictive variable (Figure 4). As Figure 4 displays, in both the training and validation cohorts, the AUCs of the developed nomogram for the 3-, 5-, and 10-year CSS were higher than those of each independent prognostic factor, indicating that the nomogram had a better precision accuracy of CSS in patients with malignant adrenal tumors. Additionally, the DCA curves demonstrated that the nomogram had a high probability of clinical applicability and might be a straightforward and efficient tool for clinical practitioners to use to assist them in better decision-making (Figure 5).

### 3.4. New CSS risk classification system

**Figure 6:**
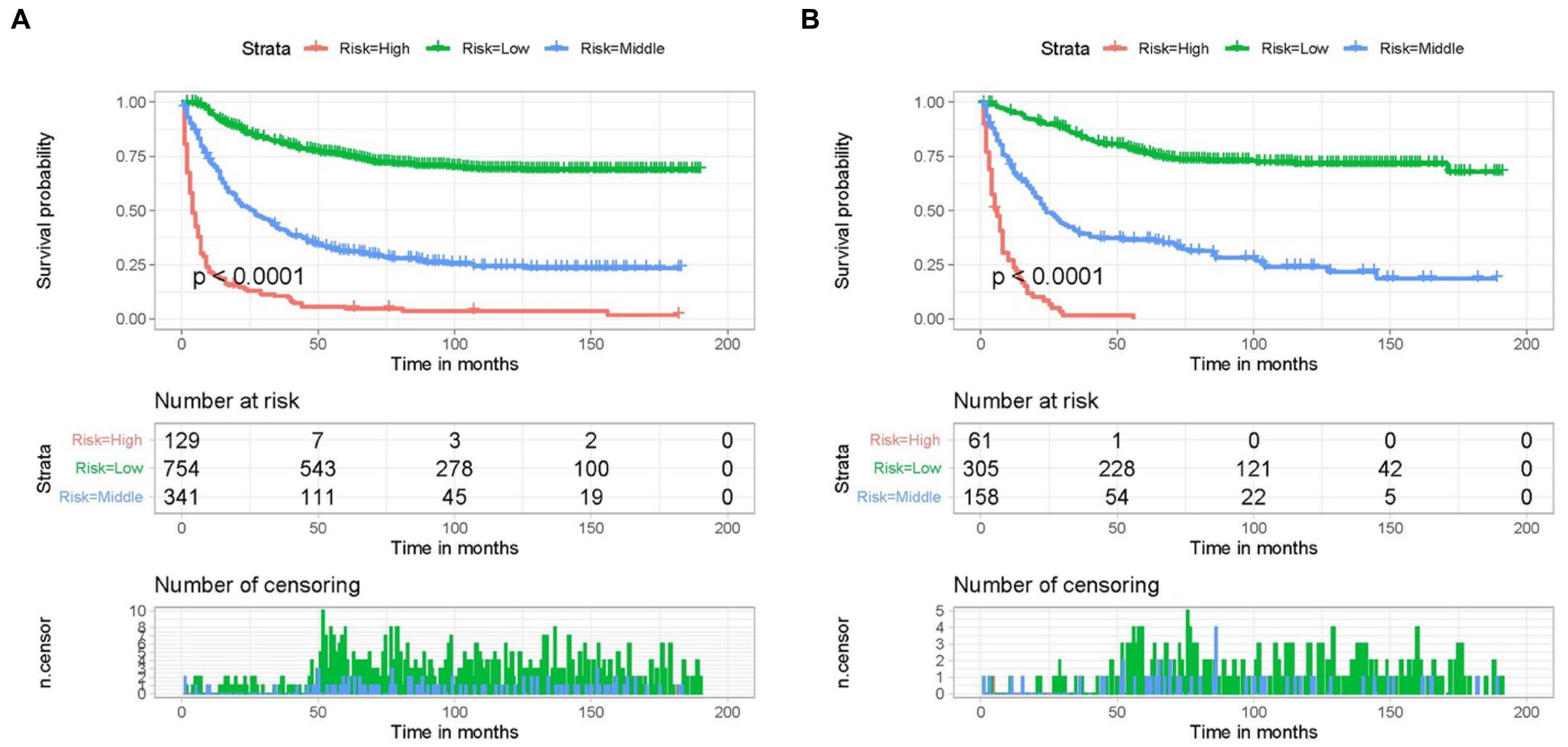
Kaplan–Meier CSS analysis of different subgroups of adrenal malignancies patients in the training **(A)** cohort and validation **(B)** cohort.

A new risk classification system needs to be developed to provide personalized management to each patient. According to the nomogram, a risk classification system was created for patients with adrenal tumors, and the classification standard was the total score obtained from the nomogram. The point values of each independent prognostic indicator were added together to establish the patient’s overall score. Using X-tile software, the optimal thresholds for the overall score were 208 and 276. The patients were categorized into three subgroups due to the overall risk score as follows: high- (>276), middle- (208–276), and low-risk (<208) groups. The KM curves also showed significant differences in CSS between the three risk groups of adrenal malignancy patients classified by the new classification system (Figure 6). As a result, the nomogram could successfully distinguish the prognosis for the various patient categories, facilitating customized patient treatment.

## 4. Discussions

Many studies have been conducted on the prognosis, diagnosis, and treatment of malignant adrenal tumors. Researchers have found that patient age, the initial stage of diagnosis, and surgical resection might affect the prognosis of patients with adrenal malignancies (6, 21, 22). However, most studies limited the tumor to a single histological type or only studied one prognostic factor and only the OS of patients. CSS offers more accurate therapy recommendations than OS and has a stronger link to tumor-mediated patient prognosis. Therefore, we have chosen CSS as the endpoint of our study. Our pre-study selected patients with primary malignant adrenal tumors from the SEER database, which showed that 603 of 5,142 patients were dead from non-cancer-specific causes and might influence the outcomes of the prognostic factors in patients. Therefore, to recognize the independent prognostic factors related to CSS outcomes, we analyzed malignant adrenal tumor subjects on the bias of an extensive population using the SEER database and built a nomogram to anticipate CSS for patients. Herein, the constructed nomogram can integrate important predictive variables and balance the effect between them, producing a quantified prediction of CSS (23). Furthermore, a nomogram-based risk classification system was established, which provides clinicians with better management of malignant adrenal tumor patients.

Our study selected 1748 patients with malignant adrenal tumors from the SEER database. Age, tumor size, histological type, tumor stage, and surgery were among the independent prognostic factors of patients with adrenal tumors discovered by the multivariate Cox regression analysis. Then, a nomogram was designated to anticipate patients’ 3-, 5-, and 10-year CSS. The survival rates of the training and validation cohorts did not show significant variation, revealing the significant discriminative power of the nomogram, as well as its prediction accuracy. Based on the five independent prognostic indicators above, a risk classification system design helped categorize the mortality risk. Based on the overall score, the subjects were divided into low-(< 208), middle- (208–276), and high- (> 276) risk groups. Each group exhibited a significant difference (*p* < 0.001).

The age of diagnosis is a vital prognostic factor for the OS of adrenal tumor patients, and older patients usually have a poor prognosis (24–26). The poor prognosis was probably due to the fact that older patients would be prone to decreased physical function, dysfunction, malnutrition, and concomitant conditions, including diabetes and hypertension, earlier relapse, and aggressive tumors resistant to multimodal and cytotoxic therapies (27, 28). Our study also found that age was one of the independent predictive variables, and individuals over 61 had a considerably poor prognosis. However, sex and race were not CSS-related variables (*p* > 0.05) in the univariate and multivariate Cox analysis, supporting earlier findings.

Tumor size and stage were probable variables that affected the OS in adrenal tumor patients (21, 29–31). Wang et al. stated that primary tumor size is a vital prognostic indicator for NB patients, and tumor size >4 cm may anticipate a poor prognosis (29). Hue et al. reported that ACC patients with tumors ≥5 cm were correlated to a high conversion rate and a subsequent elevation in margin positivity, thus having a poor prognosis (30). Furthermore, many reports revealed that patients with distant metastasis have significantly lower OS than those with localized or regional tumors (21, 31, 32). The results of our study supported the previous reports that distant metastasis and larger tumor size (63–111 mm, > 111 mm) exhibited worse CSS in patients with adrenal malignancies. These patterns revealed the necessity for early detection and intervention to prolong patient survival and reduce the incidence of tumor recurrence and metastasis. Additionally, our study indicated that the histological type was an independent prognostic predictor of malignant adrenal tumors. The prognosis of the patient whose tumor histological type was ACC was the worst, while NB patients had a better prognosis, and pheochromocytoma had the best prognosis out of all the histological types.

Surgical resection of tumors is still an effective treatment for malignant adrenal tumors, and many researchers have validated its therapeutic effect. Patients with localized tumors are recommended to have surgical resection of the tumor in conjunction with the removal of lymph nodes adjacent to the tumor (33). If more than 90% of the tumor and all the metastases can be removed for patients with regional or metastatic tumors, resection may be an option (34). After undergoing systemic treatment, surgery may be considered again in some circumstances, such as when the bulky disease is extensive or when less than 90% of the tumor is accessible for removal. Adjuvant therapy can be considered if the patient is at high risk for local recurrence and tumor metastasis based on positive margins, ruptured capsule, large tumor size, and high grade (35). In these cases, adjuvant RT with an external beam to the tumor bed was a possibility; this was primarily done if there was a concern of tumor spillage or positive margins following surgery (36). Adjuvant chemotherapy can also be an option following the primary tumor resection or for patients with metastases, even though its application in this circumstance is debatable. Our study demonstrated that patients who received surgery showed a better prognosis, while radiotherapy, chemotherapy, and surgery/radiation sequence were not independent prognostic factors for malignant adrenal tumor patients.

Despite all these advantages, including the construction of a nomogram with a good prediction and risk classification performance, this research has several restrictions: (i) the retrospective clinical study would inevitably include selection bias, (ii) some diagnostic and treatment data were unknown, such as the molecular signatures, patient radiotherapy dose and regimen, adverse effects, and so on, and (iii) the multicenter clinical validation has temporarily been unassessed regarding the external efficacy of our nomogram.

## 5. Conclusion

Malignant adrenal tumor patients with age > 61, tumor size >111 mm, distant metastases, and ACC as histological type had a poor prognosis. Patients who received surgery had a good prognosis. Developing an efficient prognostic nomogram and mortality risk stratification system helped anticipate 3-, 5-, and 10-year CSS for adrenal tumor patients, helping clinicians make the correct decisions of clinical practice and optimizing therapeutic benefits.

## Data availability statement

The dataset from the SEER database that was generated and/or analyzed during the current study is available in the SEER dataset repository (https://seer.cancer.gov/). Data downloading and processing are as described in Method. All data and materials in the study are available from the corresponding author upon reasonable request.

## Ethics statement

All methods were carried out in accordance with relevant guidelines and regulations. Data extraction and usage have been approved by SEER Program. All the data can be found in the SEER dataset: https://seer.cancer.gov/seerstat/. We obtained access to the SEER database after obtaining permission to access research data files with the reference number 17720-Nov2021.

## Author contributions

ML designed the study and wrote the main manuscript. XD extracted the data, did the analysis, and prepared figures. DY and LL provided critical comments and revised the manuscript. All authors contributed to the article and approved the submitted version.

## Funding

This work was supported by the Jilin Provincial Department of Science and Technology (grant number: YDZJ202102CXJD020).

## Conflict of interest

The authors declare that the research was conducted in the absence of any commercial or financial relationships that could be construed as a potential conflict of interest.

## Publisher’s note

All claims expressed in this article are solely those of the authors and do not necessarily represent those of their affiliated organizations, or those of the publisher, the editors and the reviewers. Any product that may be evaluated in this article, or claim that may be made by its manufacturer, is not guaranteed or endorsed by the publisher.

## Footnotes

^1^ https://seer.cancer.gov/seerstat/

## Abbreviations

CSS: cancer-specific survival
ROC: receiver operating characteristic
DCA: decision curve analysis
AUC: area under the curve
AI: adrenal incidentalomas
ACC: adrenocortical carcinoma
NB: neuroblastoma
OS: overall survival
SEER: Surveillance, Epidemiology and End Results
PHEO: pheochromocytoma

## References

[1] Kim JH Choi MH. Embryonic development and adult regeneration of the adrenal gland. Endocrinol Metab. (2020) 35:765–73. doi: 10.3803/EnM.2020.403, PMID: 33397037 PMC7803617

[2] Lee JM Kim MK Ko SH Koh JM Kim BY Kim SW. Clinical guidelines for the Management of Adrenal Incidentaloma. Endocrinol Metab. (2017) 32:200–18. doi: 10.3803/EnM.2017.32.2.200, PMID: 28685511 PMC5503865

[3] Mayer SK Oligny LL Deal C Yazbeck S Gagné N Blanchard H. Childhood adrenocortical tumors: case series and reevaluation of prognosis--a 24-year experience. J Pediatr Surg. (1997) 32:911–5. doi: 10.1016/S0022-3468(97)90649-7, PMID: 9200099

[4] Boland GW Blake MA Hahn PF Mayo-Smith WW. Incidental adrenal lesions: principles, techniques, and algorithms for imaging characterization. Radiology. (2008) 249:756–75. doi: 10.1148/radiol.2493070976, PMID: 19011181

[5] Sherlock M Scarsbrook A Abbas A Fraser S Limumpornpetch P Dineen R. Adrenal Incidentaloma. Endocr Rev. (2020) 41:775–820. doi: 10.1210/endrev/bnaa008, PMID: 32266384 PMC7431180

[6] Ahmed AA Thomas AJ Ganeshan DM Blair KJ Lall C Lee JT. Adrenal cortical carcinoma: pathology, genomics, prognosis, imaging features, and mimics with impact on management. Abdom Radiol. (2020) 45:945–63. doi: 10.1007/s00261-019-02371-y, PMID: 31894378

[7] Thampi A Shah E Elshimy G Correa R. Adrenocortical carcinoma: a literature review. Transl Cancer Res. (2020) 9:1253–64. doi: 10.21037/tcr.2019.12.28, PMID: 35117470 PMC8797314

[8] Angelousi A Kyriakopoulos G Athanasouli F Dimitriadi A Kassi E Aggeli C. The role of Immunohistochemical markers for the diagnosis and prognosis of adrenocortical neoplasms. J Pers Med. (2021) 11:208. doi: 10.3390/jpm11030208, PMID: 33804047 PMC8001501

[9] Gatta G van der Zwan JM Casali PG Siesling S Dei Tos AP Kunkler I. Rare cancers are not so rare: the rare cancer burden in Europe. Eur J Cancer. (2011) 47:2493–511. doi: 10.1016/j.ejca.2011.08.008 22033323

[10] Coughlan D Gianferante M Lynch CF Stevens JL Harlan LC. Treatment and survival of childhood neuroblastoma: evidence from a population-based study in the United States. Pediatr Hematol Oncol. (2017) 34:320–30. doi: 10.1080/08880018.2017.1373315, PMID: 29039999 PMC6764456

[11] Rickman DS Schulte JH Eilers M. The expanding world of N-MYC-driven tumors. Cancer Discov. (2018) 8:150–63. doi: 10.1158/2159-8290.CD-17-0273, PMID: 29358508

[12] Whittle SB Smith V Doherty E Zhao S McCarty S Zage PE. Overview and recent advances in the treatment of neuroblastoma. Expert Rev Anticancer Ther. (2017) 17:369–86. doi: 10.1080/14737140.2017.1285230, PMID: 28142287

[13] Wienke J Dierselhuis MP Tytgat GAM Künkele A Nierkens S Molenaar JJ. The immune landscape of neuroblastoma: challenges and opportunities for novel therapeutic strategies in pediatric oncology. Eur J Cancer. (2021) 144:123–50. doi: 10.1016/j.ejca.2020.11.014, PMID: 33341446

[14] Hayes G. Update on adrenalectomy. Vet Clin North Am Small Anim Pract. (2022) 52:473–87. doi: 10.1016/j.cvsm.2021.12.005, PMID: 35210060

[15] Vaidya A Nehs M Kilbridge K. Treatment of adrenocortical carcinoma. Surg Pathol Clin. (2019) 12:997–1006. doi: 10.1016/j.path.2019.08.010 31672303

[16] Guo Q Wang Y An J Wang S Dong X Zhao H. A prognostic model for patients with gastric signet ring cell carcinoma. Technol Cancer Res Treat. (2021) 20:15330338211027912. doi: 10.1177/15330338211027912 34190015 PMC8258759

[17] Mohler JL Antonarakis ES Armstrong AJ D’Amico AV Davis BJ Dorff T. Prostate Cancer, version 2.2019, NCCN clinical practice guidelines in oncology. J Natl Compr Cancer Netw. (2019) 17:479–505. doi: 10.6004/jnccn.2019.0023, PMID: 31085757

[18] Huang C He J Ding Z Li H Zhou Z Shi X. A nomogram for predicting the risk of bone metastasis in newly diagnosed head and neck Cancer patients: a real-world data retrospective cohort study from SEER database. Front Genet. (2022) 13:865418. doi: 10.3389/fgene.2022.865418, PMID: 35706444 PMC9189363

[19] Mathew G Agha R Albrecht J Goel P Mukherjee I Pai P. STROCSS 2021: strengthening the reporting of cohort, cross-sectional and case-control studies in surgery. Ann Med Surg (Lond). (2021) 72:103026. doi: 10.1016/j.amsu.2021.103026, PMID: 34820121 PMC8599107

[20] Zhang J Yang W Lian C Zhao Q Ming WK Ip CC. A nomogram for predicting survival in patients with skin non-keratinizing large cell squamous cell carcinoma: a study based on the surveillance, epidemiology, and end results database. Front Med. (2023) 10:1082402. doi: 10.3389/fmed.2023.1082402, PMID: 36873873 PMC9983752

[21] Lv Z Yu Y Luo Y Lin S Xiang X Mao X. Long-term survival outcomes of pediatric adrenal malignancies: an analysis with the upstaged SEER registry during 2000-2019. Front Endocrinol. (2022) 13:977105. doi: 10.3389/fendo.2022.977105, PMID: 36171902 PMC9511147

[22] Xie W Zhang Y Cao R. Construction and validation of a prognostic model for predicting overall survival of primary adrenal malignant tumor patients: a population-based study with 1,080 patients. Front Surg. (2022) 9:1025213. doi: 10.3389/fsurg.2022.1025213, PMID: 36353609 PMC9637840

[23] Fakhry C Zhang Q Nguyen-Tân PF Rosenthal DI Weber RS Lambert L. Development and validation of nomograms predictive of overall and progression-free survival in patients with oropharyngeal Cancer. J Clin Oncol. (2017) 35:4057–65. doi: 10.1200/JCO.2016.72.0748, PMID: 28777690 PMC5736236

[24] Ahmed AA Zhang L Reddivalla N Hetherington M. Neuroblastoma in children: update on clinicopathologic and genetic prognostic factors. Pediatr Hematol Oncol. (2017) 34:165–85. doi: 10.1080/08880018.2017.1330375, PMID: 28662353

[25] Sun Q Chen Y Jin Q Yuan X. A nomogram for predicting recurrence-free survival of intermediate and high-risk neuroblastoma. Eur J Pediatr. (2022) 181:4135–47. doi: 10.1007/s00431-022-04617-2, PMID: 36149505

[26] Mizdrak M Tičinović Kurir T Božić J. The role of biomarkers in adrenocortical carcinoma: a review of current evidence and future perspectives. Biomedicine. (2021) 9:174. doi: 10.3390/biomedicines9020174, PMID: 33578890 PMC7916711

[27] Liu Q Feng L Xue H Su W Li G. Development and validation of a nomogram to predict the overall survival of patients with neuroblastoma. Medicine (Baltimore). (2020) 99:e19199. doi: 10.1097/MD.0000000000019199, PMID: 32150058 PMC7478547

[28] Burdett N Vincent AD O’Callaghan M Kichenadasse G. Competing risks in older patients with Cancer: a systematic review of geriatric oncology trials. J Natl Cancer Inst. (2018) 110:825–30. doi: 10.1093/jnci/djy111, PMID: 30011032

[29] Wang J-X Cao ZY Wang CX Zhang HY Fan FL Zhang J. Prognostic impact of tumor size on patients with neuroblastoma in a SEER-based study. Cancer Med. (2022) 11:2779–89. doi: 10.1002/cam4.4653, PMID: 35315591 PMC9302263

[30] Hue JJ Bingmer K Zhao H Ammori JB Wilhelm SM Towe CW. Reassessing the impact of tumor size on operative approach in adrenocortical carcinoma. J Surg Oncol. (2021) 123:1238–45. doi: 10.1002/jso.26418, PMID: 33577722

[31] Miao J Wei H Cui J Zhang Q Liu F Mao Z. The prognosis of different distant metastases pattern in malignant tumors of the adrenal glands: a population-based retrospective study. PLoS One. (2022) 17:e0264431. doi: 10.1371/journal.pone.0264431, PMID: 35290387 PMC8923449

[32] Yan P Qi F Bian L Xu Y Zhou J Hu J. Comparison of incidence and outcomes of neuroblastoma in children, adolescents, and adults in the United States: a surveillance, epidemiology, and end results (SEER) program population study. Med Sci Monit. (2020) 26:e927218. doi: 10.12659/MSM.927218 33249420 PMC7711874

[33] Autorino R Bove P de Sio M Miano R Micali S Cindolo L. Open versus laparoscopic adrenalectomy for adrenocortical carcinoma: a Meta-analysis of surgical and oncological outcomes. Ann Surg Oncol. (2016) 23:1195–202. doi: 10.1245/s10434-015-4900-x, PMID: 26480850

[34] Silvinato A Bernardo WM Branco AW. Total and partial laparoscopic adrenalectomy. Rev Assoc Med Bras (1992). (2019) 65:1240. doi: 10.1590/1806-9282.65.10.1240, PMID: 31721954

[35] Shah MH Goldner WS Benson AB Bergsland E Blaszkowsky LS Brock P. Neuroendocrine and adrenal tumors, version 2.2021, NCCN clinical practice guidelines in oncology. J Natl Compr Cancer Netw. (2021) 19:839–68. doi: 10.6004/jnccn.2021.0032, PMID: 34340212

[36] Zhu J Zheng Z Shen J Lian X Miao Z Shen J. Efficacy of adjuvant radiotherapy for treatment of adrenocortical carcinoma: a retrospective study and an updated meta-analysis. Radiat Oncol. (2020) 15:118. doi: 10.1186/s13014-020-01533-3, PMID: 32448148 PMC7245885

